# Supplementary material for: Electrospun Nanofiber Dopped with TiO2 and Carbon Quantum Dots for the Photocatalytic Degradation of Antibiotics
Source: Polymers (Basel). 2024 Oct 22;16(21):2960. doi: 10.3390/polym16212960 (PMC11548584; doi:10.3390/polym16212960)
Supplement: Supplementary file 1 [file polymers-16-02960-s001.zip › polymers-3257211-supplementary.pdf]

Supplementary material

Eletrospun nanofiber dopped with TiO_2 and carbon quantum dots for the photocatalytic degradation of antibiotics

Valentina Silva [1], Diana L. D. Lima [2], Etelvina de Matos Gomes [3], Bernardo Almeida [3], Vânia Calisto [1], Rosa M. F. Baptista [3,*], and Goreti Pereira [1,*]

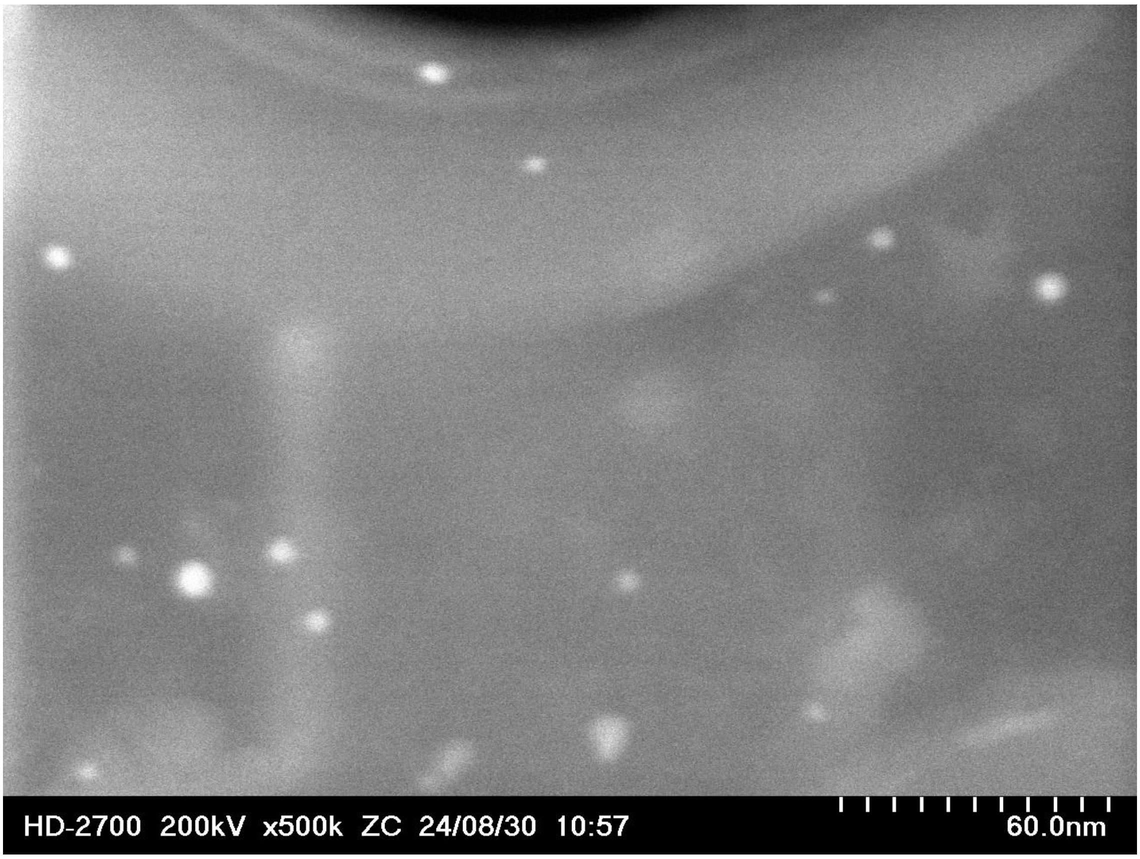

Figure S1. TEM image of carbon quantum dots.
